# Functional redundancy of PI3K isoforms in conventional T cells provides a selective Treg-targeting strategy through inhibition of PI3K-delta isoform

**DOI:** 10.1186/2051-1426-2-S3-O4

**Published:** 2014-11-06

**Authors:** Shamim Ahmad, Mikayel Mkrtichyan, Rasha Abu Eid, Rajeev Shrimali, Atbin Doroodchi, Samir N Khleif

**Affiliations:** 1 Georgia Regents University Cancer Center, Augusta, Georgia, United States; 2 Georgia Regents University, Augusta, Georgia, United States

## Introduction

Increased regulatory T cell (Treg) numbers within tumors and circulation of cancer patients, observed in early studies, implied their involvement in pathogenesis and disease progression. Also, Treg increase in cancer patients have been associated with reduced survival and inhibition of anti-tumor immune responses. Therefore, decreasing the numbers and/or function of Tregs is needed to facilitate better outcomes for cancer patients. The phosphoinositide 3-kinase (PI3K-Akt) pathway plays important roles in cell growth, survival, and proliferation of T cells. However, little is known about the role of different isoforms of PI3K in different T subsets activation. Here, we explore the role of PI3K isoforms in Tregs and conventional T cell activation with the intention of identifying potential differences to selectively inhibit Tregs.

## Methods

For *in vitro *analysis, FACS-sorted Tregs and Tconvs from Foxp3-GFP mice were stimulated (anti-CD3 Ab/anti-CD28 Ab/IL-2) with or without inhibitors for 72 hrs. Phosphorylation of Akt (S473) and S6 was analyzed by flow. Violet Cell Trace (VCT) proliferation assay was performed using flow cytometry. The *in vivo *effect of inhibitors on frequency of different T cell subsets were assessed in TC-1 tumor-bearing mice on days 3 and 6 after treatment.

## Results

We found that pharmacologic inhibition of PI3Kδ isoform but not PI3Kα or PI3Kβ exhibit a selective blockade of PI3K/Akt signaling and the inhibition of proliferation of Tregs. In contrast, the inhibition of each individual Class IA PI3K isoform in conventional T cells did not affect their activation and proliferation. Further, our data on combined inhibition of PI3K isoforms suggest that these isoforms are redundant and compensate for each other in Tconvs. Interestingly, PI3Kα or PI3Kβ were unable to compensate for PI3Kδ in Tregs, making PI3Kδ inhibition a selective Treg-targeting approach. Importantly, we also show that inhibition of PI3Kδ and not PI3Kα PI3Kb in tumor-bearing mice significantly decreases the level of Tregs without affecting other T cell subsets. We believe that our findings provide a basis for development of novel cancer immunotherapies based on selective inhibition of Tregs.

